# Computational Insights into Membrane Disruption by Cell-Penetrating Peptides

**DOI:** 10.1021/acs.jcim.4c01940

**Published:** 2025-01-17

**Authors:** Eric Catalina-Hernandez, Marcel Aguilella-Arzo, Alex Peralvarez-Marin, Mario Lopez-Martin

**Affiliations:** † Unit of Biophysics, Department of Biochemistry and Molecular Biology, Facultat de Medicina, Av. Can Domènech s/n, Universitat Autònoma de Barcelona, 08193 Cerdanyola del Vallès, Catalonia, Spain; ‡ Institute of Neurosciences, Universitat Autònoma de Barcelona, 08193 Cerdanyola del Vallès, Catalonia, Spain; § Laboratory of Molecular Biophysics, Department of Physics, University Jaume I, 12071 Castellon, Spain

## Abstract

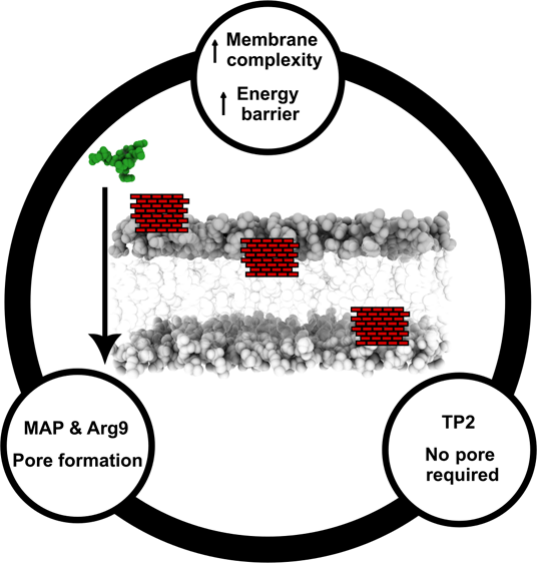

Cell-penetrating peptides (CPPs) can translocate into cells without inducing cytotoxicity. The internalization process implies several steps at different time scales ranging from microseconds to minutes. We combine adaptive Steered Molecular Dynamics (aSMD) with conventional Molecular Dynamics (cMD) to observe nonequilibrium and equilibrium states to study the early mechanisms of peptide–bilayer interaction leading to CPPs internalization. We define three membrane compositions representing bilayer sections, neutral lipids (i.e., upper leaflet), neutral lipids with cholesterol (i.e., hydrophobic core), and neutral/negatively charged lipids with cholesterol (i.e., lower leaflet) to study the energy barriers and disruption mechanisms of Arg9, MAP, and TP2, representing cationic, amphiphilic, and hydrophobic CPPs, respectively. Cholesterol and negatively charged lipids increase the energetic barriers for the peptide–bilayer crossing. TP2 interacts with the bilayer by hydrophobic insertion, while Arg9 disrupts the bilayer by forming transient or stable pores. MAP has shown both behaviors. Collectively, these findings underscore the significance of innovative computational approaches in studying membrane-disruptive peptides and, more specifically, in harnessing their potential for cell penetration.

## Introduction

The lipid fraction of biological membranes is mostly composed of phospholipids, which accounts for selective permeation, such as the cell membrane, a highly selective and dynamic barrier that encloses the contents of all living cells, responsible for cellular structural integrity and intra- and extracellular homeostasis. Cell-penetrating peptides (CPPs) are small peptides that can be found in nature and are capable of efficiently crossing the cell membrane. CPPs’ optimal and efficient design to transport cargo molecules into the cell is of paramount importance.^1,2^ CPPs have emerged as powerful tools with promising outcomes in fields such as drug delivery,^3^ diagnosis of diseases,^4^ and therapeutics.^5^ For instance, CPPs have been used as therapeutic agents targeting specific cell types,^6^ or coupled with anticancer molecules targeting tumor tissue, while healthy tissue remains unharmed.^7−9^

CPPs translocate across cellular membranes via diverse mechanisms that can be classified into energy-independent and energy-dependent pathways.^10^ Energy-dependent translocation involves three types of endocytosis, namely, macropinocytosis, caveolae-mediated, and clathrin-mediated endocytosis.^11^ Energy-independent penetration includes the pore formation,^12^ the carpet-like model (through membrane destabilization without pore formation),^13^ the membrane thinning model,^14^ and inverted micelle formation.^15^ However, direct validation of these energy-independent models has only been obtained for inverted micelles,^16^ and the other translocation methods have not yet been completely described.

Based on their physicochemical properties, CPPs have been classified^17^ into cationic, such as nona-arginine (Arg9);^18^ hydrophobic, such as Kaposi fibroblast growth factor (K-FGF)^19^ or Translocating peptide 2 (TP2);^20^ and amphipathic, such as Transportan 10 (TP10)^21^ or model amphipathic peptides (MAP), a group of peptides derived from the α-helical amphipathic model peptide, designed in 1991, and here referred to as MAP.^22,23^ Besides, amphipathic CPPs can be further divided as primary amphipathic (defined by their hydrophobic domains), secondary amphipathic (forming α-helices with one hydrophilic and one hydrophobic faces), β-sheet (that have a hydrophobic stretch and a hydrophilic stretch), proline-rich, and histidine-rich.^20,24^ Therapeutic applications of these CPPs include their use in drug delivery, anticancer or anti-inflammatory treatments, among others.^19,25−29^ Nonetheless, CPPs encounter limitations such as instability, since they are prone to proteolytic degradation; lack of selectivity, which could provoke toxicity or side effects and limited efficacy, given that some CPPs only show powerful penetrating activity at high micromolar concentrations (>10 μM).^30^ From the computational perspective, translocation of any CPP is a relatively slow process and computationally too demanding to be observed in a conventional molecular dynamics (cMD) simulation.^79^ In this study, we examine the membrane disruption potential as an important step of the internalization process. We use adaptive steered molecular dynamics (aSMD) by applying an external potential followed by cMD to assess whether an equilibrium has been reached (i.e., the CPPs have overcome the bilayer energy barrier to cross) or not, as well as to analyze the bilayer-peptide interactions of CPPs. In order to represent the three main blocks, we decided to study a cationic CPP (Arg9), a hydrophobic CPP (TP2), and an amphipathic CPP (MAP).

## Models and Methods

### Systems Preparation

Peptides were initially modeled with ColabFold notebook,^32^ using AlphaFold^33^ model for monomer prediction, and were relaxed in an explicit solvent system at 310.15 K. AMBER20 program was used to perform the simulations.^34^ The AMBER ff14SB^35^ force field and periodic boundary conditions were applied, and the SHAKE algorithm^36^ was used to restrain the hydrogen atoms, allowing for a 2 fs time step. Besides, the Monte Carlo method was used to add 150 mM KCl ions and water TIP3P molecules to solvate the system. A short minimization (5000 cycles) and NVT equilibration (125 ps) were run with a restraint force of 1 kcal·mol^–1^·Å^–2^ on the peptide, before the unrestrained cMD simulation of 100 ns.

A peptide–bilayer system was built in CHARMM-GUI^37−43^ for each relaxed peptide and membrane composition combination, amounting for a total of 12 systems (3 control membranes, without peptide, 1 for each bilayer, plus 9 peptide systems: 3 membrane compositions for 3 peptides). Here, a single peptide was placed approximately 10 Å from the center of mass (COM) of the upper leaflet bilayer membrane. The N-terminus or C-terminus of the peptides was not modified to any extent.

Three symmetric membrane compositions were defined. First, one is constituted of 1,2-dipalmitoylphosphatidylcholine (DPPC), a neutral, simple bilayer model commonly used in biophysical studies. Besides, it has been used in previous CPPs studies^44^ and can be used to compare the results obtained. Second, following the same study,^44^ we also used a more complex membrane, namely, DPPC:DOPC:CHOL, where DOPC stands for dioleoylphosphatidylcholine and CHOL for cholesterol, with the addition of cholesterol and a lipid with an unsaturated tail. Third, we expanded the study of CPP behavior by adding negatively charged lipids, that is, DPPC:DOPC:DPPS:DOPS:CHOL membrane, where DPPS stands for dipalmitoylphosphatidylserine and DOPS for Dioleoylphosphatidylserine–. To avoid bias, the molar ratio of lipids was kept balanced in the 2 heterogeneous bilayer systems. Moreover, to avoid membrane deformation artifacts in this pulling experiment, we used 150 lipids per leaflet which, according to Hub et al.^45−47^ prevents such artifacts since the bilayers are large enough. The exact composition of each membrane is the following: DPPC (150 DPPC lipids); DPPC:DOPC:CHOL (50:50:50 lipids, respectively); DPPC:DOPC:DPPS:DOPS:CHOL (30:30:30:30:30 lipids, respectively). The same conditions as those in the peptide relaxing simulations were used. For the membrane lipids, the Amber Lipid21^48^ force field was selected.

Thereafter, the systems were energy minimized for 5,000 steps and equilibrated during 3.5 ns, starting in the NVT ensemble with positional restraints on the membrane atoms (restraint force of 2.5 kcal·mol^–1^·Å^–2^), and changing to the NPT ensemble after 500 ps while lowering the positional restraints on the membrane throughout the NPT equilibration procedure (1, 0.5, 0.2, and 0 kcal·mol^–1^·Å^–2^, respectively). Lastly, the membrane was relaxed for 100 ns of conventional molecular dynamics. During this step, the peptide was kept restrained to avoid peptide–membrane interaction and allow for an unperturbed membrane relaxation (restraint force of 10 kcal·mol^–1^·Å^–2^).

### Adaptive Steered Molecular Dynamics (aSMD)

Peptide translocation is a procedure computationally too expensive to observe in a conventional molecular dynamics simulation, as it commonly occurs in the scale of seconds to minutes.^79^ Consequently, we accelerated that process by using steered molecular dynamics (SMD).^49^ SMD is a molecular dynamics enhanced sampling method where an external potential is applied to accelerate the movement of a specific group of atoms, in this case, the peptide, along a defined set of coordinates. The *z* direction, the membrane normal direction, was defined as the pulling coordinate of the peptide. The reaction coordinate was defined as the distance between the COM of the carbon alpha (CA) residues of the peptide and the COM of the lipids’ polar head in the lower part of the bilayer, namely, phosphate, nitrogen, oxygen, and the three main carbon atoms of this group.

In SMD simulations, many simulations must be run to achieve convergence of the potential of mean force (PMF). Adaptive steered molecular dynamics (aSMD)^50,51^ was introduced to alleviate this problem. In aSMD, the reaction coordinate, here, the distance between the COM of peptide’s CA atoms and membrane lower leaflets polar head’s COM, is divided into different steps. Then, separate SMD simulations are performed in each of these stages. In this case, the membrane length (ca. 40 Å) was divided into 8 stages of 5 Å and 25 replicas were run for each step (with a constant force of 10 kcal·mol^–1^), thus using aSMD, as utilized in previous studies.^44,52−54^ Briefly, after each step, the Jarzynski average^55−57^ across all replicas was calculated, and the last frame of the closest replica was used as input for the following step. Each aSMD step was run at 1 Å per nanosecond (5 ns per replica), discussed below. An aSMD step totaled 125 ns per step and 1000 ns per aSMD simulation. Altogether, ∼9 μs was run for the aSMD simulations of all 3 peptides.

**Figure 1:**
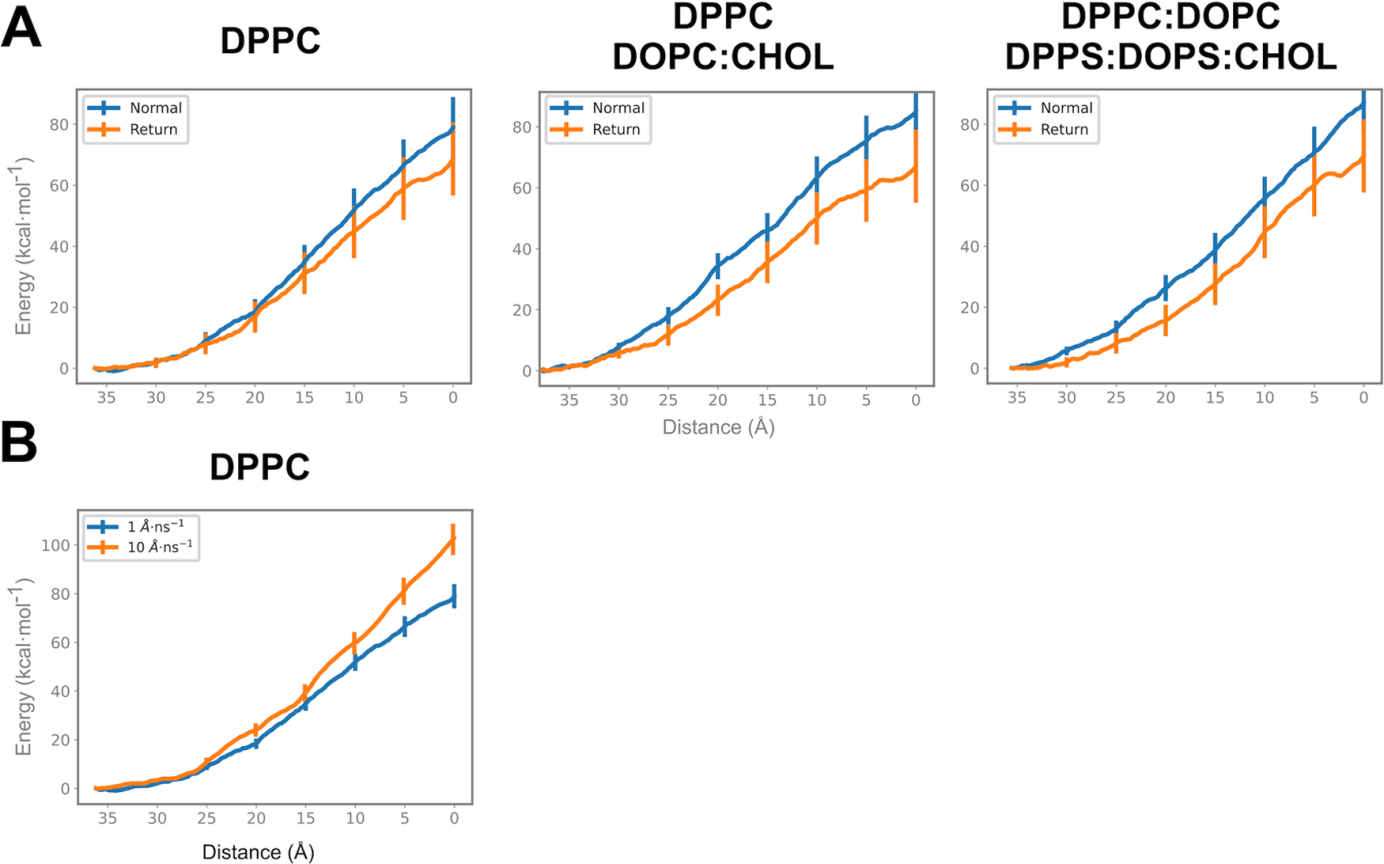
Potential of mean force (PMF) calculation in (A) forward and backward aSMD simulations and (B) different pulling speed simulations. (A) PMF has been calculated forward (normal, blue) and backward (return, orange) to calibrate the system and test that the bilayers’ energy barriers were comparable. The effect of normal and return aSMD has been computed for the three membrane compositions. (B) To test the best pulling velocity, aSMD has been simulated at two different speeds: 10 Å/ns (orange) and 1 Å/ns (blue). The effect of pulling speed has been done in DPPC membrane.

To calibrate the system for aSMD and to determine that the membrane bilayer systems were comparable in terms of energy barrier, we performed a set of forward–backward simulations in all three bilayer systems using a single Arg residue (Arg1, Figure 1A). The reaction coordinate used was the same as in previously described aSMD simulations (distance between COM of the peptide’s CA atom and COM of the lipids’ polar head in the lower part of the bilayer). Forward and backward PMF values for Arg1 are within the same energy interval; thus, the model membranes are valid to be used in this study. It is important to state that the higher the heterogenicity in the bilayer composition, the higher the differences in the elastic/viscoelastic behavior in the forward/backward pathways, as happens in real biological systems. In parallel, in order to choose the pulling velocity, aSMD simulations were performed at different pulling speeds. Park and Schulten studied SMD with two pulling velocities: 100 and 10 Å/ns. Since they concluded that the lower the pulling velocity, the more accurate the PMF calculation,^57,58^ we decided to use 10 Å/ns. Besides, we compared it to the velocity used in more recent studies,^44,59^ 1 Å/ns. Therefore, the pulling speeds chosen are 10 and 1 Å/ns (Figure 1B). The results show that with a slower velocity, the lipids had more time to adjust, leading to a lower and more accurate PMF.^57,58^ Consequently, we decided to use the slowest pulling speed (1 Å/ns) for subsequent simulations.

### PMF Calculation

The Potential of the Mean Force is computed by employing the Jarzynski equality.^56^ The Jarzynski equality is a powerful relationship that connects the nonequilibrium work performed during SMD simulations to the free energy difference between two states (A and B), as seen in eq 1:
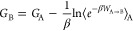
1where β is the Boltzmann constant multiplied by the temperature (k_B_·T) and the tangled brackets indicate averaging over multiple trajectories.

In this study, after each aSMD step, the replica with the closest work value to the Jarzynski average was selected as the starting point for the next simulation step. This approach helps remove the trajectories that minimally contribute to the overall PMF and significantly reduces the number of simulations required for convergence.^34^

### Conventional Molecular Dynamics (cMD)

Lastly, starting from the last frame of the aSMD simulation last step (where the distance between peptide and lower leaflet COMs is 0 Å), a 100 ns unbiased cMD (also referred to as the *relaxation step*) was run with the purpose of allowing the system to relax after an external potential addition. The same simulating conditions were used as in the previous cases. A total of ∼3 μs were run for the final relaxation part, accounting for 100 ns for each of the simulations (100 ns × 3 peptides × 3 membrane compositions × 3 replicas). Besides, the 3 control systems (without peptide) were run following the same equilibration and production protocol.

### Data Analysis

Trajectory visual analysis was performed with Visual Molecular Dynamics (VMD),^60^ CPPTraj, and PyTraj.^61^ PyLipID^62^ and LiPyPhilic^63−66^ were used to analyze the simulations. An in-house script was used to analyze the lipid order parameter. Lipid order parameter, typically denoted as S_CD_, measures the orientation of the C–D bond in lipid acyl chains relative to the bilayer normal.^67^ It is calculated using eq 2:
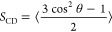
2where θ is the (time-dependent) angle between the C–D bond and a reference axis. The angular brackets represent an ensemble average over time and lipid molecules. Lipid order parameter values closer to 0 indicate complete disorder and closer to 1 indicate perfect alignment along the bilayer normal. An in-house Python script was implemented to compute the pore size distribution, calculating the minimum pore size on the *z* axis of the membrane. This script calculates the maximum distance of the water residues per each membrane *z*-stack and outputs the minimum distance of all of the *z*-stacks per each simulation frame. Matplotlib^68^ and Seaborn^69^ were used for graphics plotting, and UCSF ChimeraX^70,71^ for molecular graphics. For the membrane analyses, only the last 80 ns of the cMD simulation was taken into account.

For benchmarking purposes, all simulations and analyses have been performed in a single GPU-based (RTX-3080Ti) workstation, running at an average of 80 ns/day accounting for a total of 150 days of computation time.

## Results and Discussion

### Bilayer Resistance to Steered Peptide Crossing

**Figure 2:**
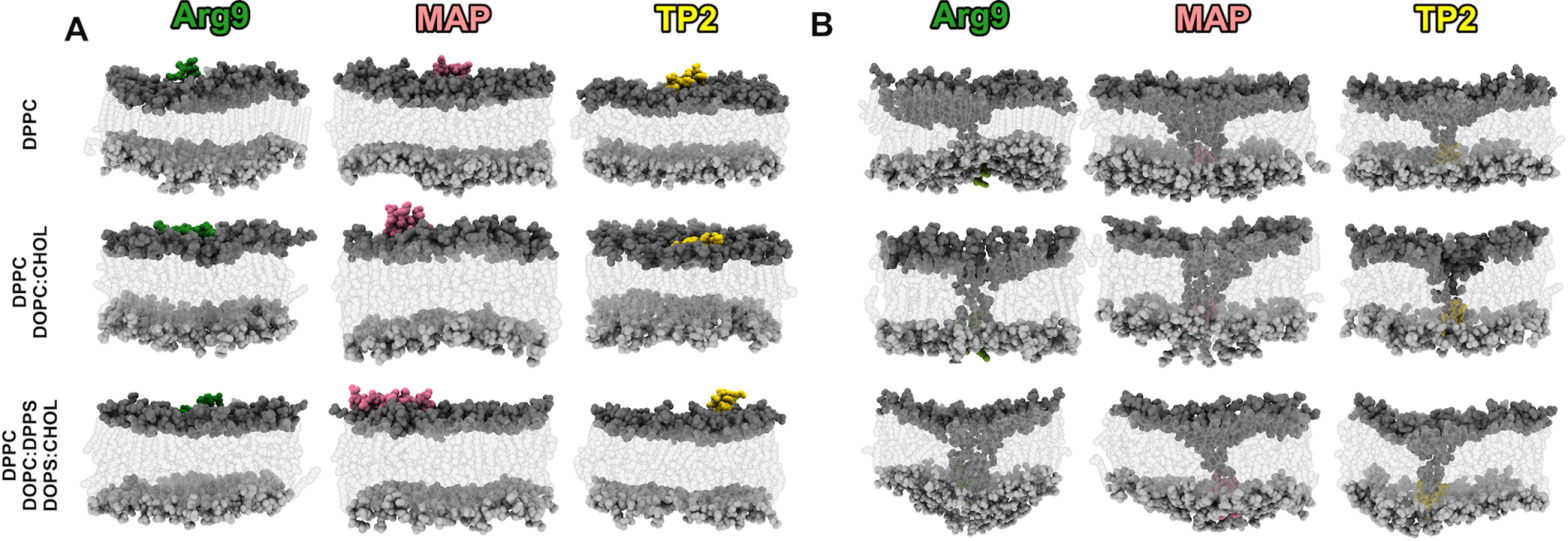
Initial and final snapshots of the aSMD process. Starting (A) and final (B) snapshots of the aSMD for the three CPPs and the three membrane compositions. See Video S1 for further details.

The simulation protocol includes two sets of simulations: aSMD for 40 ns divided into 8 steps and 25 replicas per step to move the peptide across the bilayer defining a nonequilibrium state, followed by 3 replicas of the relaxation step, consisting of 100 ns of cMD each. This experimental design was applied to investigate the behavior of 3 canonical CPPs (Arg9, MAP, and TP2, see Table 1) in 3 different membranes (Figure 2A). As a simplification of a complex cellular bilayer, a CPP, when internalized into the cell, first encounters the extracellular leaflet, rich in neutral polar headgroups, which can be related to the DPPC system. Second, the CPP enters in contact with the hydrophobic core of the bilayer, with cholesterol and unsaturated lipid tails, as in the DPPC:DOPC:CHOL system. Third, the CPP needs to break the interaction with the hydrophobic core and interact with the intracellular leaflet, richer in negatively charged polar headgroups, as in the DPPC:DOPC:DPPS:DOPS:CHOL system. In short, we have modeled simplified systems for each bilayer phase, with the DPPC system being equivalent to the extracellular leaflet, DPPC:DOPC:CHOL to the hydrophobic core, and DPPC:DOPC:DPPS:DOPS:CHOL to the intracellular leaflet, respectively.

**Table 1:** Characteristics of the Peptides Used in This Study. GRAVY Score Is Calculated from Ref (72)

**peptide**	**length**	**sequence**	**type**	**net charge**	**GRAVY score**
**Arg9**	9	RRRRRRRRR	cationic	+9	–4.5
**MAP**	18	KLALKLALKALKAALKLA	amphipathic	+5	0.99
**TP10**	21	AGYLLGKINLKALAALAKKIL	amphipathic	+4	0.93
**TP2**	13	PLIYLRLLRGQWC	hydrophobic	+2	0.42
**K-FGF**	17	AAVALLPAVLLALLAP	hydrophobic	0	2.42

After the aSMD simulation, the molecular distributions are similar for all cases (Figure 2B): the peptide has been steered into the lower part of the bilayer and is in contact with the polar heads of the lipids in the lower part of the bilayer. Some polar heads of the upper leaflet have been dragged along with the peptide during the steering process, in agreement with the previously described “Defect Assisted by Charge” (DAC) phenomenon,^73^ and the polar heads of the upper bilayer contact those of the lower bilayer. As seen in Figure 2B, on average, MAP causes the highest membrane disturbance, and TP2 causes the lowest membrane disturbance (DAC). This means that the DAC caused is related, but not directly proportional, to the peptide charge, as discussed by Elber.^73^ The author stated that, when working with a CPP, there is a higher number of degrees of freedom and charge plays a lesser role. Conversely, for small molecules, charge plays an important part in the DAC created. Besides, for CPPs, the peptide length seems to be an important aspect since MAP (18 residues, net charge +5) produces more DAC than Arg9 (9 residues, net charge +9) even though it has a smaller net charge.

**Figure 3:**
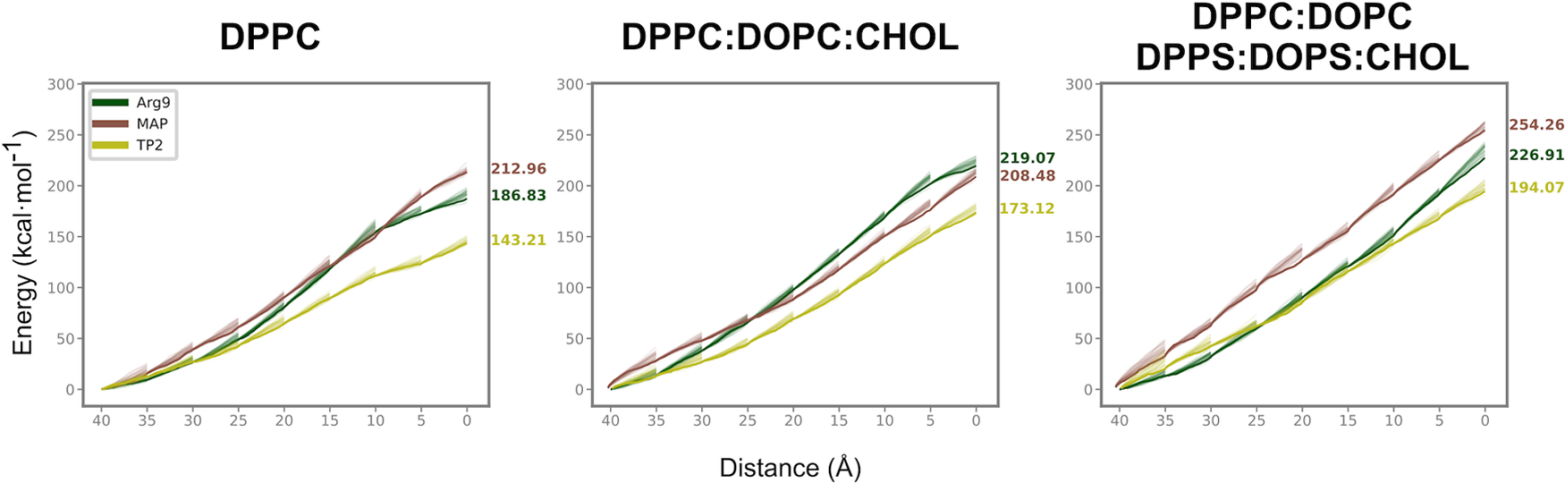
PMF barrier of peptides with respect to the membrane composition. The values indicated correspond to the last value (highest energy) of the PMF analysis. PMF profiles of the three membrane compositions are shown. PMF profiles of all replicas are shown with a transparency of 10%.

PMF values are indicative of the resistance opposed by the bilayer during the peptide crossing, showing that bilayer complexity is, on average, positively correlated to higher PMF values (Figure 3). In the DPPC membrane, peptides exhibit, on average, the lowest energy requirement to traverse the bilayer, indicated by a mean PMF barrier of 181.52 ± 20.33 kcal·mol^–1^. The introduction of cholesterol to the membrane results in an overall increase in the mean PMF barrier, to 200.91 ± 13.87 kcal·mol^–1^. Cholesterol has been associated with reduced efficiency in CPP translocation, a phenomenon previously discussed by Pae et al.^74^ Addition of unsaturated fatty acids (DOPC) should enhance the internalization of CPPs and lower the PMF,^75^ but this effect seems to be counterbalanced by the influence of cholesterol. Finally, in the DPPC:DOPC:DPPS:DOPS:CHOL membrane, we observe the highest resistance to bilayer crossing, with a mean PMF barrier of 225.65 ± 17.40 kcal·mol^–1^. This PMF increase can be related to the effect of increased adsorption in the upper leaflet when negative lipids are present,^76^ requiring higher energy to break these lipid–peptide interactions.

Arg9 in DPPC and in DPPC:DOPC:CHOL shows a similar PMF value (∼190 and ∼220 kcal·mol^–1^, respectively, as seen in Figure 3) to previously published data.^44^ Besides, the energy required to move Arg9 into the middle of the DPPC membrane (from 40 to 20 Å) is similar to the energy obtained in a previous study using neutral lipids.^77^ Taking into consideration all three CPPs and the three bilayer systems (Figure 3), TP2 and Arg9 partition more efficiently in the upper leaflet (DPPC) compared with MAP. The transition energy from the water-bilayer interface to the hydrophobic core (DPPC to DPPC:DOPC:CHOL) is lower for MAP and TP2, and slightly higher for Arg9. Finally, from the hydrophobic core to the lower leaflet (DPPC:DOPC:DPPS:DOPS:CHOL) all peptides require higher energy for the transition, especially MAP.

aSMD has demonstrated PMF value accuracy calculation for peptides^50−52,78^ and the relative trends shown for the peptides studied here are qualitatively coherent and considered as a measure to compare each peptide in the three bilayer compositions. This is of paramount importance in CPPs, where sequences differ significantly in amino acid composition, secondary structure propensities, length, and physicochemical properties. Thus, the quantitative assessment of PMF values should be interpreted with caution. For absolute quantitative output, computationally demanding methods with higher sampling such as multibranched aSMD (MB-ASMD), full-relaxation aSMD (FR-ASMD),^52^ or adaptively biasing MD (ABMD),^44^ should be considered to obtain fully converging PMF profiles,^79^ although the different nature among peptides should still pose a limitation.

### Peptide Release after aSMD

**Figure 4:**
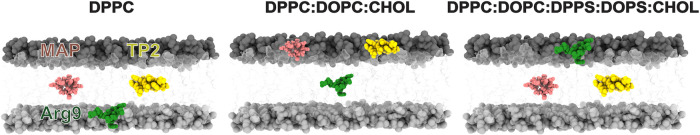
Illustrative representation of the peptide location in the 3 membrane compositions after the 100 ns of conventional MD (relaxation). Peptides are colored as Arg9 in dark green, MAP in rose, TP2 in gold. The polar heads of phospholipids in both the upper and lower bilayers are illustrated in darker and lighter shades of gray, respectively, while the lipid tails are portrayed in transparent white. Peptide colors are maintained in the following figures. Waters are omitted for clarity. See Video S1 for further details.

At the end of the aSMD simulations, the peptide has been successfully transferred to the lower region of the lipid bilayer. It is important to determine whether this steered process has overcome the bilayer energy barrier, reaching an equilibrium state (the energy of the process has been released) or not (the energy of the process is stored in the last step of the aSMD simulation). Thus, we performed three replicas (all with the same outcome) of cMD simulations relaxing the system to compare the peptides’ behavior in each bilayer system (Video S1). At this stage we observed four possible behaviors for the peptides: (1) “Lower leaflet equilibrium state”: after the aSMD simulation, the peptide has reached an energy minimum and stays at the lower part of the bilayer; (2) “Pore formation”: the energy stored in the process results in the peptide bouncing back toward the upper leaflet remaining in the hydrophobic core and leading to formation of pores of different radius in the membrane; we define a pore as a large defect in the membrane that allows for a continuous water flow between the upper and lower leaflets; (3) “Insertion”: the energy stored in the process results in the peptide bouncing back toward the upper leaflet remaining in the hydrophobic core of the bilayer without leading to pore formation; (4) “Return”: the energy stored in the process results in the peptide bouncing back to the upper part of the bilayer. For the sake of clarity, a summary of these behaviors, observed across all peptides and membrane compositions, is presented in Figure 4 and Table 2.

**Table 2:** Simulation Results for All CPPs in the 3 Membrane Compositions^a^

		**DPPC**	**DPPC:DOPC**
**peptide**	**DPPC**	**DOPC:CHOL**	**DPPS:DOPS:CHOL**
Arg9	lower leaflet equilibrium state	large pore	return
MAP	small pore	return	insertion
TP2	insertion	return	insertion

a All replicas show the same behavior, and the ratios are thus omitted for clarity. See Table 3 for small or large pore details.

In the cMD simulation, Arg9 overcomes the imposed DPPC bilayer energy barrier since it stays in the lower leaflet for 100 ns of simulation (equilibrium state), although the formation of a small transient pore is observed (Table 3 and Figure S1). The cMD simulation for Arg9 in DPPC:DOPC:CHOL shows a relaxation from a nonequilibrium state to a more stable state where Arg9 remains trapped in the bilayer hydrophobic core while forming a large-sized pore (Table 3 and Figure S1 for pore details). In the DPPC:DOPC:DPPS:DOPS:CHOL membrane, the energy stored at the end of the Arg9 aSMD simulation is sufficient to return the peptide back to the upper leaflet.

**Table 3:** Mean Radius Size (Å) of the Last 80 ns of the Relaxation

**peptide**	**DPPC**	**DPPC:DOPC:CHOL**	**DPPC:DOPC:DPPS:DOPS:CHOL**
**Arg9**	0.19 ± 0.03	6.30 ± 0.04	0
**MAP**	0.71 ± 0.03	0	0
**TP2**	0	0	0

The cMD simulation for MAP in DPPC shows the peptide bouncing back but remaining in the hydrophobic core of the bilayer, forming a small pore. The cMD simulation for MAP in DPPC:DOPC:CHOL shows a relaxation of the peptide and an upper part reallocation. In the DPPC:DOPC:DPPS:DOPS:CHOL bilayer, the cMD simulation for MAP shows how the peptide returns to the upper bilayer but becomes inserted into the hydrophobic core. In average, MAP has the highest PMF values, indicating that an internalization process is not as favorable as in the other cases. This can be related to experiments where they observed that the internalization of MAP requires, in a large amount, an energy-dependent pathway or vesicle transport event.^22,80−82^

Similarly to MAP, TP2 has not reached an equilibrium in the lower part of the bilayer under any condition. In DPPC:DOPC:CHOL, TP2 releases all of the stored energy and returns to the upper bilayer, indicating that cholesterol-induced rigidity poses a high energy barrier for TP2 to remain in the bilayer. On the other hand, in DPPC and DPPC:DOPC:DPPS:DOPS:CHOL bilayers, we observe the insertion of TP2 in the hydrophobic core of the bilayer, but without leading to the formation of a pore. This behavior can be related to the fact that TP2 in monomeric form enters the cell via spontaneous membrane translocation, rather than the pore formation mechanism.^83,84^

Effects of the peptides on bilayer behavior have been performed, namely, lipid order parameter, membrane thickness, and area per lipid (Figure S1). Membrane thickness and area per lipid fluctuate accordingly. DPPC membrane has the lowest area per lipid (∼60.1 Å^2^ is the average value for all peptides over the simulation) and membrane thickness (average value of ∼38.5 Å along the simulation), indicating that DPPC is the most compact membrane. The addition of cholesterol has been documented to decrease area per lipid,^85^ but it seems that the addition of unsaturated lipids (DOPC) counterbalances cholesterol’s effect due to the kinks in its structure, and makes the bilayer less compact, showcasing higher area per lipid (average of ∼75 Å^2^) and membrane thickness (∼41.6 Å). Third, the addition of negatively charged lipids compacts the membrane (thickness of ∼40.8 Å), while lowering area per lipid (∼65.6 Å^2^). Area per lipid and membrane thickness analyses can also be related to the fluctuations in PMF among membranes. First, DPPC has the lowest average PMF value. DPPC:DOPC:CHOL is less compact, which should lower PMF values, but this effect is counterbalanced by cholesterol, which does not favor peptide crossing, as previously discussed. In DPPC:DOPC:DPPS:DOPS:CHOL membrane, negatively charged lipids tighten the membrane and strengthen interactions between peptide and membrane,^76^ causing the highest increase in PMF values.

**Figure 5:**
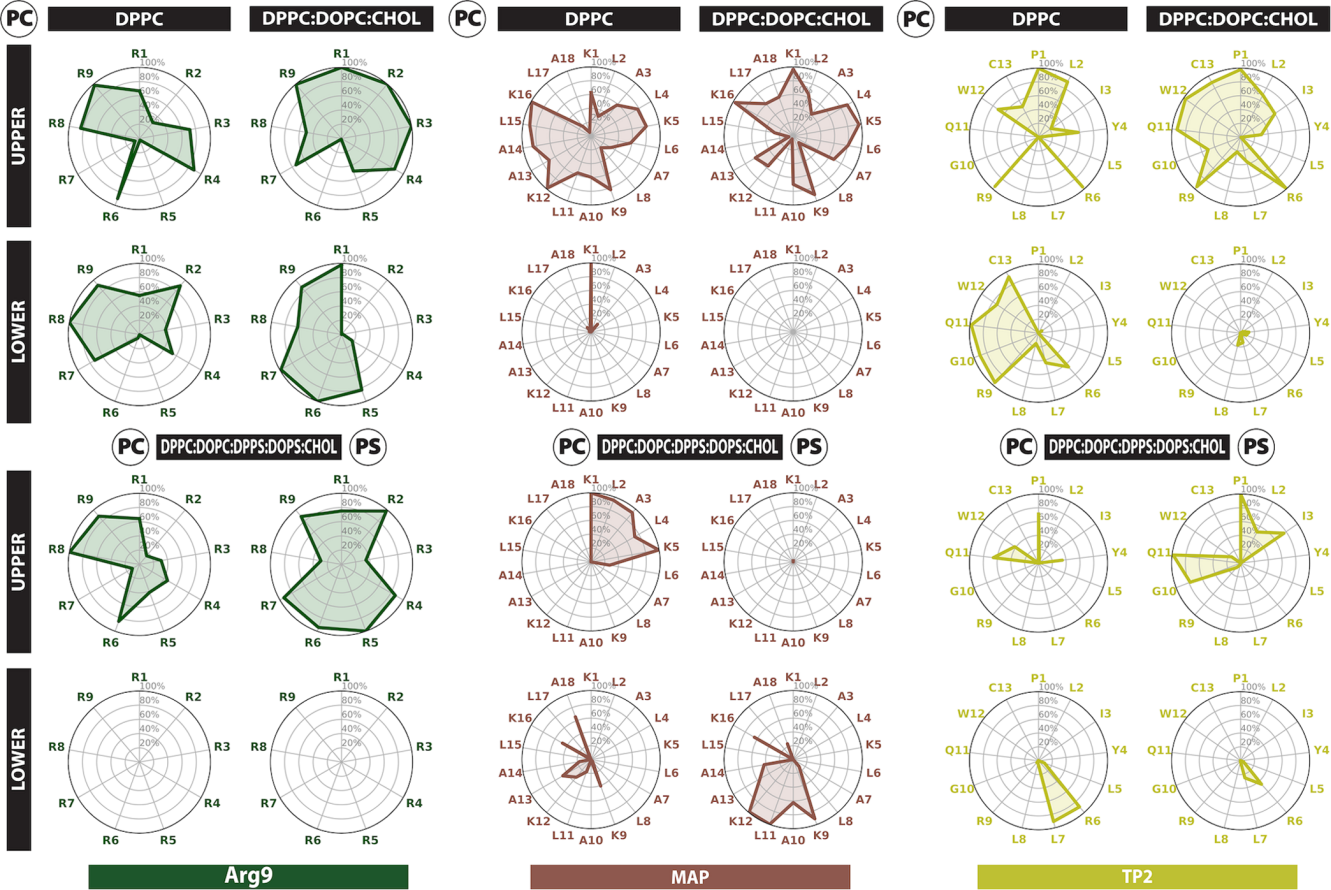
Residue occupancy by the polar head of the phospholipids in the upper and lower leaflets. Polar heads corresponding to PC (phosphatidylcholine) occupancy is shown for DPPC, DPPC:DOPC:CHOL, and DPPC:DOPC:DPPS:DOPSCHOL membranes. For the third membrane, PS (phosphatidylserine) occupancy is also shown. The occupancy analysis refers to the first replica.

Lipid order parameter values are in line with previously reported values,^86^ showing that membranes are well organized, thus indicating that the CPPs do not destabilize the membrane upon interaction and/or disruption. Furthermore, secondary structure analyses were conducted; however, the peptides do not exhibit any defined secondary structure (Figure S2). Thus, we focused on the occupancy of peptide residues by the polar heads of the phospholipids in upper and lower leaflets (Figure 5).

### Peptide–Bilayer Interactions

Sequence composition, charge, and the hydrophobicity index GRAVY score (Table 1) are key determinants driving peptide–bilayer interactions. The GRAVY score is a measure of peptide hydrophobicity based on the Kyte-Doolittle scale,^87^ where the more negative the value the higher peptide hydrophilicity, and the more positive the value the higher the hydrophobicity. In this regard, Arg9 has a highly negative GRAVY score, indicating how Arg9 is more likely to interact with water and, in this case, with lipids’ polar heads. Thus, Arg9 shows 3 different modes: lower bilayer steady-state, upper part relocation, or pore formation, but, in each of these, it stays in contact with the polar heads of the bilayer (in the two former cases) or with the waters (in the latter case). In the other two canonical CPPs, MAP and TP2, we see how they get inserted into the bilayer and stay in contact with the hydrophobic part of the membrane, which is related to the more hydrophobic nature indicated by the GRAVY score. The differences in GRAVY score explain why MAP and TP2 can get inserted into the bilayer without pore formation, but Arg9 requires to be in contact with water and forces pore formation. In parallel, both MAP and TP2 have key positively charged residues (Table 1), which allows them to interact with the polar heads in the membrane.

In Figure 5, we present the occupancy analysis regarding the lipids’ polar heads for every peptide in all membranes, such as PC for phosphatidylcholine (in DPPC/DOPC) and PS for phosphatidylserine (in DPPS/DOPS). Occupancy is defined as the percentage of simulation time that a residue is in contact with a lipid. In Figure S3, we show occupancy by the lipid tails and cholesterol. Regarding peptide-polar head interactions (Figure 5), we observe a higher interaction ratio for Arg9 (several residues have close to 100% occupancy), which can be explained due to the polycationic nature of this CPP, strongly attracted to the negatively charged polar heads of the lipids. K/R neighboring residues also show high occupancy in all three CPPs. MAP, which has alternating positive (K) and hydrophobic (L, A) residues, preferably interacts with the polar heads through positive residues, that is, K1, K5, K9, K12, and K16. TP2 contains only two charged residues, R6 and R9, which are prone to interact with the polar heads of the lipids and show high occupancy across the three bilayers. However, the N- and C-terminal parts also interact with the polar heads in three and two bilayers, respectively. In DPPC, the peptide is inserted into the membrane and stretched, thus interacting with a leaflet in each end. In the DPPC:DOPC:CHOL bilayer, R9 favors the lipid interaction of the TP2 C-terminal residues. Besides, the N-terminal residues (especially P1) show high occupancy, which can be explained by the positive charge in the N-terminal residue. On the other hand, regarding the occupancies by lipid tails, Arg9 shows, on average, low occupancy, again explained by its polycationic nature, whereas MAP and TP2 show high occupancy by the lipid tails, mainly interacting with the hydrophobic residues (L, A in MAP, and L, I, Y, and W in TP2).

In parallel, when comparing the occupancies across all three bilayers, there are noteworthy differences between: (1) the case where the peptide has reached an equilibrium in the lower part of the bilayer, which has a higher occupancy in the lower leaflet (Arg9 in DPPC), (2) the peptides that form a pore and interact with the polar heads in upper and lower leaflets (MAP in DPPC, and Arg9 in DPPC:DOPC:CHOL), (3) the peptides that get inserted into the bilayer and also interact with both leaflets (TP2 in DPPC, MAP and TP2 in DPPC:DOPC:DPPS:DOPS:CHOL), and (4) the peptides that have been reallocated to the upper leaflet and are only interacting with the polar heads in the upper leaflet (MAP and TP2 in DPPC:DOPC:CHOL, Arg9 in DPPC:DOPC:DPPS:DOPS:CHOL). MAP in the first membrane composition generates a pore in the bilayer, and only interacts with the lower leaflet with the first residue, indicating that it shows an extended conformation, perpendicular to the membrane,^88,89^ stabilized by the hydrophobic interactions with the lipid tails and the hydrophilic interactions with water, with a similar distribution to the three cases of insertion (see Video S1). Interestingly, Arg9 in DPPC:DOPC:DPPS:DOPS:CHOL interacts rather with the polar heads in PS lipids than PC lipids, likely by the strong attraction between the side chains and the negatively charged lipids, as seen in previous studies.^90^

In short, specific phospholipid preferences can be extracted from this study. Arg9 has a preference for polar heads, and if both PC and PS heads are present, then Arg9 favors the interaction with PS lipids. TP2 and MAP have a higher interaction with lipid tails but also interact strongly with PC heads, indicating that they have parts with a preference for heads and other parts that prefer to interact with lipid tails. No preference for cholesterol interaction has been seen, similar to the results seen for other CPPs.^91^

The cationic Arg9 seems to require pore formation to cross the bilayer^92,93^ as we observe for Arg9 by either forming transient (in the DPPC bilayer) or more stable (in DPPC:DOPC:CHOL) pores, likely a mechanism to overcome the bilayer energy barriers. For MAP, the energy barrier could be lowered by the formation of a pore,^94^ related to what we see in DPPC, but MAP also seems to require translocation through energy-dependent mechanisms.^82^ TP2 may involve direct translocation (through a quick and transient pore or without pore formation as we observe here) of a monomeric peptide,^83,84^ leading to a minimum leakage.

There are other aspects, beyond the scope of this study, that could play a relevant role in the internalization process of CPPs and would require further investigation, such as secondary structure conversions, peptide organization, and/or peptide self-assembly.^73,77,90,95,96^

## Conclusions

In conclusion, we have analyzed the effect of neutral, saturated, and unsaturated lipids, cholesterol, and negatively charged lipids on the membrane disturbing potential of representative CPPs. As a general conclusion, the presence of cholesterol adds more stability to the membrane and increases thickness, which entails higher deformation resistance. Negatively charged lipids are not directly correlated to the internalization efficiency of CPPs. CPPs interactions with the upper leaflet strongly influence the ability of the peptides to interact with the lower leaflet and, consequently, their ability to form pores or reach the lower leaflet. In cationic CPPs, such as Arg9, the peptide–lipid and peptide–water interactions lead to a larger disturbance of the bilayer and formation of large transient pores which would be key to overcome the energy barrier at the hydrophobic core layer. Hydrophobic CPPs, such as TP2, find a lower energy transition path across the bilayer without requiring the formation of transient pores. Amphiphilic CPPs, such as MAP, find a limiting step in the upper leaflet partitioning, requiring the formation of transient water pores to overcome energetic barriers that are opposed by the bilayer.

In this study, due to computing restraints, we have focused on a representative peptide of the three main CPP groups (cationic, amphipathic, and hydrophobic) and studied against three simplified model membranes to understand CPPs membrane disruption capacity. Further studies should consider the plethora of CPPs available, their physicochemical properties, translocation mechanisms, and specific lipid–peptide interactions in biological membranes. Better characterization and understanding of the diverse CPP mechanism are of paramount importance, which will lead to the more efficient design and development of CPPs and their cargoes. Enhancing the CPPs targeting and internalization potential will lead to better and more personalized drug delivery systems, anticancer, antimicrobial, and/or antiviral therapies.^73,77,90,95,96^ Despite the significant strides made in the understanding of CPP internalization, translocation, and pore formation, certain aspects remain elusive, which underscores the need for future investigation as well as the need for out-of-the-box ideas to study such processes.

## Data Availability

Code and required files to reproduce the analysis performed here are available at: https://github.com/APMlab-memb/CPPs_aSMD_cMD.git. Due to file size limitations, the simulation trajectory files will be shared upon request.

## Supporting Information Available

Additional analyses including pore size, lipid order parameter, membrane thickness, and area per lipid analyses for all peptides in all membranes (Figure S1), secondary structure analysis (Figure S2), and occupancy of the lipid tails and cholesterols for all peptides in all membranes (Figure S3) (PDF); aSMD and cMD simulations for all peptides and membranes (MOV) (Video S1) (ZIP)

## References

[ref1] Lindgren M.; Hällbrink M.; Prochiantz A.; Langel Ü. Cell-Penetrating Peptides. Trends Pharmacol. Sci. 2000, 21 (3), 99–103. 10.1016/S0165-6147(00)01447-4. 10689363

[ref2] Schwarze S. R.; Hruska K. A.; Dowdy S. F. Protein Transduction: Unrestricted Delivery into All Cells?. Trends Cell Biol. 2000, 10 (7), 290–295. 10.1016/S0962-8924(00)01771-2. 10856932

[ref3] Tian Y.; Zhou S. Advances in Cell Penetrating Peptides and Their Functionalization of Polymeric Nanoplatforms for Drug Delivery. Wiley Interdiscip. Rev.:Nanomed. Nanobiotechnol. 2021, 13 (2), e1668 10.1002/wnan.1668. 32929866

[ref4] Tripathi P. P.; Arami H.; Banga I.; Gupta J.; Gandhi S. Cell Penetrating Peptides in Preclinical and Clinical Cancer Diagnosis and Therapy. Oncotarget 2018, 9 (98), 37252 10.18632/oncotarget.26442. 30647857 PMC6324683

[ref5] Zhang Y.; Guo P.; Ma Z.; Lu P.; Kebebe D.; Liu Z. Combination of Cell-Penetrating Peptides with Nanomaterials for the Potential Therapeutics of Central Nervous System Disorders: A Review. J. Nanobiotechnol. 2021, 19 (1), 1–22. 10.1186/s12951-021-01002-3. PMC8381574 34425832

[ref6] Li T.; Bourgeois J.-P.; Celli S.; Glacial F.; Le Sourd A.-M.; Mecheri S.; Weksler B.; Romero I.; Couraud P.-O.; Rougeon F.; Lafaye P. Cell-Penetrating Anti-GFAP VHH and Corresponding Fluorescent Fusion Protein VHH-GFP Spontaneously Cross the Blood-Brain Barrier and Specifically Recognize Astrocytes: Application to Brain Imaging. FASEB J. 2012, 26 (10), 3969–3979. 10.1096/fj.11-201384. 22730440

[ref7] Li Q.; Yang J.; Chen C.; Lin X.; Zhou M.; Zhou Z.; Huang Y. A Novel Mitochondrial Targeted Hybrid Peptide Modified HPMA Copolymers for Breast Cancer Metastasis Suppression. J. Controlled Release 2020, 325, 38–51. 10.1016/j.jconrel.2020.06.010. 32598957

[ref8] Guo F.; Fu Q.; Zhou K.; Jin C.; Wu W.; Ji X.; Yan Q.; Yang Q.; Wu D.; Li A.; Yang G. Matrix Metalloprotein-Triggered, Cell Penetrating Peptide-Modified Star-Shaped Nanoparticles for Tumor Targeting and Cancer Therapy. J. Nanobiotechnol. 2020, 18 (1), 48 10.1186/s12951-020-00595-5. PMC7076984 32183823

[ref9] Yang Y.; Yang Y.; Xie X.; Cai X.; Mei X. Preparation and Characterization of Photo-Responsive Cell-Penetrating Peptide-Mediated Nanostructured Lipid Carrier. J. Drug Targeting 2014, 22 (10), 891–900. 10.3109/1061186X.2014.940589. 25045925

[ref10] Gestin M.; Dowaidar M.; Langel Ü. Uptake Mechanism of Cell-Penetrating Peptides. In Peptides and Peptide-based Biomaterials and their Biomedical Applications; Sunna A.; Care A.; Bergquist P. L., Eds.; Advances in Experimental Medicine and Biology; Springer International Publishing: Cham, 2017; pp 255–264. 10.1007/978-3-319-66095-0_11 29081057

[ref11] Doherty G. J.; McMahon H. T. Mechanisms of Endocytosis. Annu. Rev. Biochem. 2009, 78, 857–902. 10.1146/annurev.biochem.78.081307.110540. 19317650

[ref12] Matsuzaki K.; Yoneyama S.; Murase O.; Miyajima K. Transbilayer Transport of Ions and Lipids Coupled with Mastoparan X Translocation. Biochemistry 1996, 35 (25), 8450–8456. 10.1021/bi960342a. 8679603

[ref13] Pouny Y.; Rapaport D.; Mor A.; Nicolas P.; Shai Y. Interaction of Antimicrobial Dermaseptin and Its Fluorescently Labeled Analogs with Phospholipid Membranes. Biochemistry 1992, 31 (49), 12416–12423. 10.1021/bi00164a017. 1463728

[ref14] Lee M.-T.; Hung W.-C.; Chen F.-Y.; Huang H. W. Many-Body Effect of Antimicrobial Peptides: On the Correlation Between Lipid’s Spontaneous Curvature and Pore Formation. Biophys. J. 2005, 89 (6), 4006–4016. 10.1529/biophysj.105.068080. 16150963 PMC1366966

[ref15] Derossi D.; Calvet S.; Trembleau A.; Brunissen A.; Chassaing G.; Prochiantz A. Cell Internalization of the Third Helix of the Antennapedia Homeodomain Is Receptor-Independent *. J. Biol. Chem. 1996, 271 (30), 18188–18193. 10.1074/jbc.271.30.18188. 8663410

[ref16] Berlose J.; Convert O.; Derossi D.; Brunissen A.; Chassaing G. Conformational and Associative Behaviours of the Third Helix of Antennapedia Homeodomain in Membrane-Mimetic Environments. Eur. J. Biochem. 1996, 242 (2), 372–386. 10.1111/j.1432-1033.1996.0372r.x. 8973656

[ref17] De Oliveira E. C. L.; Da Costa K. S.; Taube P. S.; Lima A. H.; Junior C. D. Biological Membrane-Penetrating Peptides: Computational Prediction and Applications. Front. Cell. Infect. Microbiol. 2022, 12, 838259 10.3389/fcimb.2022.838259. 35402305 PMC8992797

[ref18] Futaki S.; Suzuki T.; Ohashi W.; Yagami T.; Tanaka S.; Ueda K.; Sugiura Y. Arginine-Rich Peptides: An Abundant Source of Membrane-Permeable Peptides Having Potential as Carriers for Intracellular Protein Delivery*. J. Biol. Chem. 2001, 276 (8), 5836–5840. 10.1074/jbc.M007540200. 11084031

[ref19] Lin Y. Z.; Yao S. Y.; Veach R. A.; Torgerson T. R.; Hawiger J. Inhibition of Nuclear Translocation of Transcription Factor NF-Kappa B by a Synthetic Peptide Containing a Cell Membrane-Permeable Motif and Nuclear Localization Sequence. J. Biol. Chem. 1995, 270 (24), 14255–14258. 10.1074/jbc.270.24.14255. 7782278

[ref20] Milletti F. Cell-Penetrating Peptides: Classes, Origin, and Current Landscape. Drug Discovery Today 2012, 17 (15–16), 850–860. 10.1016/j.drudis.2012.03.002. 22465171

[ref21] Yandek L. E.; Pokorny A.; Florén A.; Knoelke K.; Langel Ü.; Almeida P. F. F. Mechanism of the Cell-Penetrating Peptide Transportan 10 Permeation of Lipid Bilayers. Biophys. J. 2007, 92 (7), 2434–2444. 10.1529/biophysj.106.100198. 17218466 PMC1864827

[ref22] Steiner V.; Schzr M.; B&men K. O.; Mutter M. Retention Behaviour of a Template-Assembled Synthetic Protein and Its Amphiphilic Building Blocks on Reversed-Phase Columns 1991; Vol. 586. 10.1016/0021-9673(91)80023-a 1806554

[ref23] Lindgren M.; Hällbrink M.; Prochiantz A.; Langel Ü. Cell-Penetrating Peptides. Trends Pharmacol. Sci. 2000, 21, 99–103. 10.1016/S0165-6147(00)01447-4. 10689363

[ref24] Iwasaki T.; Tokuda Y.; Kotake A.; Okada H.; Takeda S.; Kawano T.; Nakayama Y. Cellular Uptake and in Vivo Distribution of Polyhistidine Peptides. J. Controlled Release 2015, 210, 115–124. 10.1016/j.jconrel.2015.05.268. 25980622

[ref25] Zaro J. L.; Fei L.; Shen W.-C. Recombinant Peptide Constructs for Targeted Cell Penetrating Peptide-Mediated Delivery. J. Controlled Release 2012, 158 (3), 357–361. 10.1016/j.jconrel.2012.01.039. 22326404

[ref26] Pescina S.; Ostacolo C.; Gomez-Monterrey I. M.; Sala M.; Bertamino A.; Sonvico F.; Padula C.; Santi P.; Bianchera A.; Nicoli S. Cell Penetrating Peptides in Ocular Drug Delivery: State of the Art. J. Controlled Release 2018, 284, 84–102. 10.1016/j.jconrel.2018.06.023. 29913221

[ref27] Pirhaghi M.; Mamashli F.; Moosavi-Movahedi F.; Arghavani P.; Amiri A.; Davaeil B.; Mohammad-Zaheri M.; Mousavi-Jarrahi Z.; Sharma D.; Langel Ü.; Otzen D. E.; Saboury A. A. Cell-Penetrating Peptides: Promising Therapeutics and Drug-Delivery Systems for Neurodegenerative Diseases. Mol. Pharmaceutics 2024, 21 (5), 2097–2117. 10.1021/acs.molpharmaceut.3c01167. 38440998

[ref28] Nakase I.; Konishi Y.; Ueda M.; Saji H.; Futaki S. Accumulation of Arginine-Rich Cell-Penetrating Peptides in Tumors and the Potential for Anticancer Drug Delivery in Vivo. J. Controlled Release 2012, 159 (2), 181–188. 10.1016/j.jconrel.2012.01.016. 22285548

[ref29] Rothbard J. B.; Garlington S.; Lin Q.; Kirschberg T.; Kreider E.; McGrane P. L.; Wender P. A.; Khavari P. A. Conjugation of Arginine Oligomers to Cyclosporin A Facilitates Topical Delivery and Inhibition of Inflammation. Nat. Med. 2000, 6 (11), 1253–1257. 10.1038/81359. 11062537

[ref30] Kim G. C.; Cheon D. H.; Lee Y. Challenge to Overcome Current Limitations of Cell-Penetrating Peptides. In Biochimica et Biophysica Acta - Proteins and Proteomics; Elsevier B.V., 2021 10.1016/j.bbapap.2021.140604. 33453413

[ref79] Yesylevskyy S.; Marrink S. J.; Mark A. E. Alternative Mechanisms for the Interaction of the Cell-Penetrating Peptides Penetratin and the TAT Peptide with Lipid Bilayers. Biophys. J. 2009, 97 (1), 40–49. 10.1016/j.bpj.2009.03.059. 19580742 PMC2711361

[ref32] Mirdita M.; Schütze K.; Moriwaki Y.; Heo L.; Ovchinnikov S.; Steinegger M. ColabFold: Making Protein Folding Accessible to All. Nat. Methods 2022, 19 (6), 679–682. 10.1038/s41592-022-01488-1. 35637307 PMC9184281

[ref33] Jumper J.; Evans R.; Pritzel A.; Green T.; Figurnov M.; Ronneberger O.; Tunyasuvunakool K.; Bates R.; Žídek A.; Potapenko A.; Bridgland A.; Meyer C.; Kohl S. A. A.; Ballard A. J.; Cowie A.; Romera-Paredes B.; Nikolov S.; Jain R.; Adler J.; Back T.; Petersen S.; Reiman D.; Clancy E.; Zielinski M.; Steinegger M.; Pacholska M.; Berghammer T.; Bodenstein S.; Silver D.; Vinyals O.; Senior A. W.; Kavukcuoglu K.; Kohli P.; Hassabis D. Highly Accurate Protein Structure Prediction with AlphaFold. Nature 2021, 596 (7873), 583–589. 10.1038/s41586-021-03819-2. 34265844 PMC8371605

[ref34] Case D. A.; Belfon K.; Ben-Shalom I. Y.; Brozell S. R.; Cerutti D. S.; T. E. Cheatham I. I. I.; Cruzeiro V. W. D.; Darden T. A.; Duke R. E.; Giambasu G.; Gilson M. K.; Gohlke H.; Goetz A. W.; Harris R.; Izadi S.; Izmailov S. A.; Kasavajhala K.; Kovalenko A.; Krasny R.; Kurtzman T.; Lee T. S.; LeGrand S.; Li P.; Lin C.; Liu J.; Luchko T.; Luo R.; Man V.; Merz K. M.; Miao Y.; Mikhailovskii O.; Monard G.; Nguyen H.; Onufriev A.; Pan F.; Pantano S.; Qi R.; Roe D. R.; Roitberg A.; Sagui C.; Schott-Verdugo S.; Shen J.; Simmerling C. L.; Skrynnikov N. R.; Smith J.; Swails J.; Walker R. C.; Wang J.; Wilson L.; Wolf R. M.; Wu X.; Xiong Y.; Xue Y.; York D. M.; Kollman P. A. AMBER. 2020; Vol. 2020.

[ref35] Maier J. A.; Martinez C.; Kasavajhala K.; Wickstrom L.; Hauser K. E.; Simmerling C. Ff14SB: Improving the Accuracy of Protein Side Chain and Backbone Parameters from Ff99SB. J. Chem. Theory Comput. 2015, 11 (8), 3696–3713. 10.1021/acs.jctc.5b00255. 26574453 PMC4821407

[ref36] Kräutler V.; van Gunsteren W. F.; Hünenberger P. H. A Fast SHAKE Algorithm to Solve Distance Constraint Equations for Small Molecules in Molecular Dynamics Simulations. J. Comput. Chem. 2001, 22 (5), 501–508. 10.1002/1096-987X(20010415)22:5<501::AID-JCC1021>3.0.CO;2-V.

[ref37] Brooks B. R.; Brooks C. L. III; Mackerell A. D. Jr.; Nilsson L.; Petrella R. J.; Roux B.; Won Y.; Archontis G.; Bartels C.; Boresch S.; Caflisch A.; Caves L.; Cui Q.; Dinner A. R.; Feig M.; Fischer S.; Gao J.; Hodoscek M.; Im W.; Kuczera K.; Lazaridis T.; Ma J.; Ovchinnikov V.; Paci E.; Pastor R. W.; Post C. B.; Pu J. Z.; Schaefer M.; Tidor B.; Venable R. M.; Woodcock H. L.; Wu X.; Yang W.; York D. M.; Karplus M. CHARMM: The Biomolecular Simulation Program. J. Comput. Chem. 2009, 30 (10), 1545–1614. 10.1002/jcc.21287. 19444816 PMC2810661

[ref38] Lee J.; Cheng X.; Swails J. M.; Yeom M. S.; Eastman P. K.; Lemkul J. A.; Wei S.; Buckner J.; Jeong J. C.; Qi Y.; Jo S.; Pande V. S.; Case D. A.; Brooks C. L. I. I. I.; MacKerell A. D. Jr.; Klauda J. B.; Im W. CHARMM-GUI Input Generator for NAMD, GROMACS, AMBER, OpenMM, and CHARMM/OpenMM Simulations Using the CHARMM36 Additive Force Field. J. Chem. Theory Comput 2016, 12 (1), 405–413. 10.1021/acs.jctc.5b00935. 26631602 PMC4712441

[ref39] Jo S.; Kim T.; Iyer V. G.; Im W. CHARMM-GUI: A Web-Based Graphical User Interface for CHARMM. J. Comput. Chem. 2008, 29 (11), 1859–1865. 10.1002/jcc.20945. 18351591

[ref40] Lee J.; Patel D. S.; Ståhle J.; Park S.-J.; Kern N. R.; Kim S.; Lee J.; Cheng X.; Valvano M. A.; Holst O.; Knirel Y. A.; Qi Y.; Jo S.; Klauda J. B.; Widmalm G.; Im W. CHARMM-GUI Membrane Builder for Complex Biological Membrane Simulations with Glycolipids and Lipoglycans. J. Chem. Theory Comput 2019, 15 (1), 775–786. 10.1021/acs.jctc.8b01066. 30525595

[ref41] Jo S.; Lim J. B.; Klauda J. B.; Im W. CHARMM-GUI Membrane Builder for Mixed Bilayers and Its Application to Yeast Membranes. Biophys. J. 2009, 97 (1), 50–58. 10.1016/j.bpj.2009.04.013. 19580743 PMC2711372

[ref42] Lee J.; Hitzenberger M.; Rieger M.; Kern N. R.; Zacharias M.; Im W. CHARMM-GUI Supports the Amber Force Fields. J. Chem. Phys. 2020, 153 (3), 035103 10.1063/5.0012280. 32716185

[ref43] Wu E. L.; Cheng X.; Jo S.; Rui H.; Song K. C.; Dávila-Contreras E. M.; Qi Y.; Lee J.; Monje-Galvan V.; Venable R. M.; Klauda J. B.; Im W. CHARMM-GUI Membrane Builder toward Realistic Biological Membrane Simulations. J. Comput. Chem. 2014, 35 (27), 1997–2004. 10.1002/jcc.23702. 25130509 PMC4165794

[ref44] Gimenez-Dejoz J.; Numata K. Molecular Dynamics Study of the Internalization of Cell-Penetrating Peptides Containing Unnatural Amino Acids across Membranes. Nanoscale Adv. 2022, 4 (2), 397–407. 10.1039/D1NA00674F. 36132688 PMC9419563

[ref45] Hub J. S. Joint Reaction Coordinate for Computing the Free-Energy Landscape of Pore Nucleation and Pore Expansion in Lipid Membranes. J. Chem. Theory Comput 2021, 17 (2), 1229–1239. 10.1021/acs.jctc.0c01134. 33427469

[ref46] Awasthi N.; Hub J. S. Simulations of Pore Formation in Lipid Membranes: Reaction Coordinates, Convergence, Hysteresis, and Finite-Size Effects. J. Chem. Theory Comput 2016, 12 (7), 3261–3269. 10.1021/acs.jctc.6b00369. 27254744

[ref47] Hub J. S.; Awasthi N. Probing a Continuous Polar Defect: A Reaction Coordinate for Pore Formation in Lipid Membranes. J. Chem. Theory Comput 2017, 13 (5), 2352–2366. 10.1021/acs.jctc.7b00106. 28376619

[ref48] Dickson C. J.; Walker R. C.; Gould I. R. Lipid21: Complex Lipid Membrane Simulations with AMBER. J. Chem. Theory Comput 2022, 18 (3), 1726–1736. 10.1021/acs.jctc.1c01217. 35113553 PMC9007451

[ref49] Case D. A.; Cheatham T. E.; Darden T.; Gohlke H.; Luo R.; Merz K. M.; Onufriev A.; Simmerling C.; Wang B.; Woods R. J. The Amber Biomolecular Simulation Programs. J. Comput. Chem. 2005, 26 (16), 1668–1688. 10.1002/jcc.20290. 16200636 PMC1989667

[ref50] Ozer G.; Quirk S.; Hernandez R. Adaptive Steered Molecular Dynamics: Validation of the Selection Criterion and Benchmarking Energetics in Vacuum. J. Chem. Phys. 2012, 136 (21), 215104 10.1063/1.4725183. 22697572

[ref51] Ozer G.; Valecv E. F.; Quirt S.; Hernandez R. Adaptive Steered Molecular Dynamics of the Long-Distance Unfolding of Neuropeptide y. J. Chem. Theory Comput 2010, 6 (10), 3026–3038. 10.1021/ct100320g. 26616767

[ref52] Zhuang Y.; Bureau H. R.; Quirk S.; Hernandez R. Adaptive Steered Molecular Dynamics of Biomolecules. Mol. Simul. 2021, 47 (5), 408–419. 10.1080/08927022.2020.1807542.

[ref53] Liu Z.; Xu Y.; Tang P. Steered Molecular Dynamics Simulations of Na + Permeation across the Gramicidin A Channel. J. Phys. Chem. B 2006, 110 (25), 12789–12795. 10.1021/jp060688n. 16800614

[ref54] Hwang H.; Schatz G. C.; Ratner M. A. Steered Molecular Dynamics Studies of the Potential of Mean Force of a Na + or K + Ion in a Cyclic Peptide Nanotube. J. Phys. Chem. B 2006, 110 (51), 26448–26460. 10.1021/jp0657888. 17181305

[ref55] Hummer G.; Szabo A. Free Energy Reconstruction from Nonequilibrium Single-Molecule Pulling Experiments. Proc. Natl. Acad. Sci. U.S.A. 2001, 98 (7), 3658–3661. 10.1073/pnas.071034098. 11274384 PMC31107

[ref56] Jarzynski C. Nonequilibrium Equality for Free Energy Differences. Phys. Rev. Lett. 1997, 78 (14), 2690–2693. 10.1103/PhysRevLett.78.2690.

[ref57] Park S.; Schulten K. Calculating Potentials of Mean Force from Steered Molecular Dynamics Simulations. J. Chem. Phys. 2004, 120 (13), 5946–5961. 10.1063/1.1651473. 15267476

[ref58] Park S.; Khalili-Araghi F.; Tajkhorshid E.; Schulten K. Free Energy Calculation from Steered Molecular Dynamics Simulations Using Jarzynski’s Equality. J. Chem. Phys. 2003, 119 (6), 3559–3566. 10.1063/1.1590311.

[ref59] Bureau H. R.; Quirk S.; Hernandez R. The Relative Stability of Trpzip1 and Its Mutants Determined by Computation and Experiment. RSC Adv. 2020, 10 (11), 6520–6535. 10.1039/D0RA00920B. 35495997 PMC9049704

[ref60] Humphrey W.; Dalke A.; Schulten K. VMD: Visual Molecular Dynamics. J. Mol. Graphics 1996, 14 (1), 33–38. 10.1016/0263-7855(96)00018-5. 8744570

[ref61] Roe D. R.; Cheatham T. E. PTRAJ and CPPTRAJ: Software for Processing and Analysis of Molecular Dynamics Trajectory Data. J. Chem. Theory Comput. 2013, 9 (7), 3084–3095. 10.1021/ct400341p. 26583988

[ref62] Song W.; Corey R. A.; Ansell T. B.; Cassidy C. K.; Horrell M. R.; Duncan A. L.; Stansfeld P. J.; Sansom M. S. P. PyLipID: A Python Package for Analysis of Protein–Lipid Interactions from Molecular Dynamics Simulations. J. Chem. Theory Comput. 2022, 18 (2), 1188–1201. 10.1021/acs.jctc.1c00708. 35020380 PMC8830038

[ref63] Smith P.; Lorenz C. D. LiPyphilic: A Python Toolkit for the Analysis of Lipid Membrane Simulations. J. Chem. Theory Comput. 2021, 17, 5907 10.1021/acs.jctc.1c00447. 34450002

[ref64] Gowers R.; Linke M.; Barnoud J.; Reddy T.; Melo M.; Seyler S.; Domański J.; Dotson D.; Buchoux S.; Kenney I.; Beckstein O. MDAnalysis: A Python Package for the Rapid Analysis of Molecular Dynamics Simulations. In Python in Science Conference 2016; pp 98–105 10.25080/Majora-629e541a-00e.

[ref65] Michaud-Agrawal N.; Denning E. J.; Woolf T. B.; Beckstein O. MDAnalysis: A Toolkit for the Analysis of Molecular Dynamics Simulations. J. Comput. Chem. 2011, 32 (10), 2319–2327. 10.1002/jcc.21787. 21500218 PMC3144279

[ref66] Ramasubramani V.; Dice B. D.; Harper E. S.; Spellings M. P.; Anderson J. A.; Glotzer S. C. Freud: A Software Suite for High Throughput Analysis of Particle Simulation Data. Comput. Phys. Commun. 2020, 254, 107275 10.1016/j.cpc.2020.107275.

[ref67] Repáková J.; Holopainen J. M.; Morrow M. R.; McDonald M. C.; Čapková P.; Vattulainen I. Influence of DPH on the Structure and Dynamics of a DPPC Bilayer. Biophys. J. 2005, 88 (5), 3398–3410. 10.1529/biophysj.104.055533. 15722435 PMC1305487

[ref68] Hunter J. D. Matplotlib: A 2D Graphics Environment. Comput. Sci. Eng. 2007, 9 (3), 90–95. 10.1109/MCSE.2007.55.

[ref69] Waskom M. L. Seaborn: Statistical Data Visualization. J. Open Source Software 2021, 6 (60), 3021 10.21105/joss.03021.

[ref70] Pettersen E. F.; Goddard T. D.; Huang C. C.; Meng E. C.; Couch G. S.; Croll T. I.; Morris J. H.; Ferrin T. E. UCSF ChimeraX: Structure Visualization for Researchers, Educators, and Developers. Protein Sci. 2021, 30 (1), 70–82. 10.1002/pro.3943. 32881101 PMC7737788

[ref71] Goddard T. D.; Huang C. C.; Meng E. C.; Pettersen E. F.; Couch G. S.; Morris J. H.; Ferrin T. E. UCSF ChimeraX: Meeting Modern Challenges in Visualization and Analysis. Protein Sci. 2018, 27 (1), 14–25. 10.1002/pro.3235. 28710774 PMC5734306

[ref72] Stothard P. The Sequence Manipulation Suite: JavaScript Programs for Analyzing and Formatting Protein and DNA Sequences. Biotechniques 2000, 28 (6), 1102 10.2144/00286ir01. 10868275

[ref73] Elber R. Defect Formation and Peptide Permeation across Phospholipid Membranes. J. Phys. Chem. B 2023, 127, 7810 10.1021/acs.jpcb.3c04895. 37678235

[ref74] Pae J.; Säälik P.; Liivamägi L.; Lubenets D.; Arukuusk P.; Langel Ü.; Pooga M. Translocation of Cell-Penetrating Peptides across the Plasma Membrane Is Controlled by Cholesterol and Microenvironment Created by Membranous Proteins. J. Controlled Release 2014, 192, 103–113. 10.1016/j.jconrel.2014.07.002. 25016968

[ref75] Zakany F.; Mándity I. M.; Varga Z.; Panyi G.; Nagy P.; Kovacs T. Effect of the Lipid Landscape on the Efficacy of Cell-Penetrating Peptides. Cells 2023, 12 (13), 1700 10.3390/cells12131700. 37443733 PMC10340183

[ref76] Jain M.; Matysiak S. Dual Role of Anionic Lipids in Amyloid Aggregation. J. Phys. Chem. B 2024, 128, 10831 10.1021/acs.jpcb.4c05636. 39450869

[ref77] MacCallum J. L.; Bennett W. F. D.; Tieleman D. P. Transfer of Arginine into Lipid Bilayers Is Nonadditive. Biophys. J. 2011, 101 (1), 110–117. 10.1016/j.bpj.2011.05.038. 21723820 PMC3127173

[ref78] Ozer G.; Quirk S.; Hernandez R. Thermodynamics of Decaalanine Stretching in Water Obtained by Adaptive Steered Molecular Dynamics Simulations. J. Chem. Theory Comput. 2012, 8 (11), 4837–4844. 10.1021/ct300709u. 26605636

[ref80] Kenien R.; Shen W. C.; Zaro J. L. Vesicle-to-Cytosol Transport of Disulfide-Linked Cargo Mediated by an Amphipathic Cell-Penetrating Peptide. J. Drug Targeting 2012, 20 (9), 793–800. 10.3109/1061186X.2012.719899. 22994388

[ref81] Oehlke J.; Lorenz D.; Wiesner B.; Bienert M. Studies on the Cellular Uptake of Substance P and Lysine-Rich, KLA-Derived Model Peptides. J. Mol. Recognit. 2005, 18, 50–59. 10.1002/jmr.691. 15386618

[ref82] Silva S.; Kurrikoff K.; Langel Ü.; Almeida A. J.; Vale N. A Second Life for MAP, a Model Amphipathic Peptide. Int. J. Mol. Sci. 2022, 23 (15), 8322 10.3390/ijms23158322. 35955457 PMC9368858

[ref83] Marks J. R.; Placone J.; Hristova K.; Wimley W. C. Spontaneous Membrane-Translocating Peptides by Orthogonal High-Throughput Screening. J. Am. Chem. Soc. 2011, 133 (23), 8995–9004. 10.1021/ja2017416. 21545169 PMC3118567

[ref84] He J.; Kauffman W. B.; Fuselier T.; Naveen S. K.; Voss T. G.; Hristova K.; Wimley W. C. Direct Cytosolic Delivery of Polar Cargo to Cells by Spontaneous Membrane-Translocating Peptides. J. Biol. Chem. 2013, 288 (41), 29974 10.1074/jbc.M113.488312. 23983125 PMC3795295

[ref85] Regen S. L. Cholesterol’s Condensing Effect: Unpacking a Century-Old Mystery. JACS Au 2022, 2 (1), 84–91. 10.1021/jacsau.1c00493. 35098224 PMC8791060

[ref86] Vermeer L. S.; De Groot B. L.; Réat V.; Milon A.; Czaplicki J. Acyl Chain Order Parameter Profiles in Phospholipid Bilayers: Computation from Molecular Dynamics Simulations and Comparison with 2H NMR Experiments. Eur. Biophys. J. 2007, 36, 919–931. 10.1007/s00249-007-0192-9. 17598103

[ref87] Kyte J.; Doolittle R. F. A Simple Method for Displaying the Hydropathic Character of a Protein. J. Mol. Biol. 1982, 157 (1), 105–132. 10.1016/0022-2836(82)90515-0. 7108955

[ref88] Yue T.; Sun M.; Zhang S.; Ren H.; Ge B.; Huang F. How Transmembrane Peptides Insert and Orientate in Biomembranes: A Combined Experimental and Simulation Study. Phys. Chem. Chem. Phys. 2016, 18 (26), 17483–17494. 10.1039/C6CP01133K. 27302083

[ref89] Kabelka I.; Vácha R. Optimal Hydrophobicity and Reorientation of Amphiphilic Peptides Translocating through Membrane. Biophys. J. 2018, 115, 1045–1054. 10.1016/j.bpj.2018.08.012. 30177443 PMC6139821

[ref90] Herce H. D.; Garcia A. E. Molecular Dynamics Simulations Suggest a Mechanism for Translocation of the HIV-1 TAT Peptide across Lipid Membranes. Proc. Natl. Acad. Sci. U.S.A. 2007, 104 (52), 20805–20810. 10.1073/pnas.0706574105. 18093956 PMC2409222

[ref91] Yi D.; Guoming L.; Gao L.; Wei L. Interaction of Arginine Oligomer with Model Membrane. Biochem. Biophys. Res. Commun. 2007, 359 (4), 1024–1029. 10.1016/j.bbrc.2007.06.015. 17572387

[ref92] Herce H. D.; Garcia A. E.; Litt J.; Kane R. S.; Martin P.; Enrique N.; Rebolledo A.; Milesi V. Arginine-Rich Peptides Destabilize the Plasma Membrane, Consistent with a Pore Formation Translocation Mechanism of Cell-Penetrating Peptides. Biophys. J. 2009, 97 (7), 1917–1925. 10.1016/j.bpj.2009.05.066. 19804722 PMC2756373

[ref93] Choe S. Translocation of a Single Arg9 Peptide across a DOPC/DOPG(4:1) Model Membrane Using the Weighted Ensemble Method. Sci. Rep. 2023, 13 (1), 1168 10.1038/s41598-023-28493-4. 36670187 PMC9860060

[ref94] Huang K.; García A. E. Free Energy of Translocating an Arginine-Rich Cell-Penetrating Peptide across a Lipid Bilayer Suggests Pore Formation. Biophys. J. 2013, 104 (2), 412–420. 10.1016/j.bpj.2012.10.027. 23442863 PMC3552254

[ref95] He X.; Lin M.; Sha B.; Feng S.; Shi X.; Qu Z.; Xu F. Coarse-Grained Molecular Dynamics Studies of the Translocation Mechanism of Polyarginines across Asymmetric Membrane under Tension. Sci. Rep. 2015, 5, 12808 10.1038/srep12808. 26235300 PMC4522684

[ref96] Oba M.; Nagano Y.; Kato T.; Tanaka M. Secondary Structures and Cell-Penetrating Abilities of Arginine-Rich Peptide Foldamers. Sci. Rep. 2019, 9 (1), 1349 10.1038/s41598-018-38063-8. 30718681 PMC6362038

