# Supplementary material for: Computational Insights into Membrane Disruption by Cell-Penetrating Peptides
Source: J Chem Inf Model. 2025 Jan 17;65(3):1549–59. doi: 10.1021/acs.jcim.4c01940 (PMC11815844; doi:10.1021/acs.jcim.4c01940)
Supplement: Supplementary file 1 — ci4c01940_si_001.zip [file ci4c01940_si_001.zip › Catalina-Hernandez_etal_SI.pdf]

Computational Insights into Membrane Disruption by Cell-Penetrating Peptides

Eric Catalina-Hernandez [1,2], Marcel Aguilella-Arzo[3], Alex Peralvarez-Marin[1,2*], Mario Lopez-Martin[1,2*]

[1]Unit of Biophysics, Department of Biochemistry and Molecular Biology, Facultat de Medicina, Av. Can Domènech s/n, Universitat Autònoma de Barcelona; 08193 Cerdanyola del Vallès, Catalonia, Spain.

[2]Institute of Neurosciences, Universitat Autònoma de Barcelona, 08193 Cerdanyola del Vallès, Catalonia, Spain.

[3]Laboratory of Molecular Biophysics, Department of Physics, University Jaume I, 12071 Castellon, Spain.

[*] Correspondence:

M.L.-M., mario.lopez@uab.cat; Tel.: +34-93-581-1907.

A.P.-M., alex.peralvarez@uab.cat; Tel.: +34-93-581-4504.

Unit of Biophysics, Dept. of Biochemistry and Molecular Biology, Facultat de Medicina, Av. Can Domènech s/n, Universitat Autònoma de Barcelona; 08193 Cerdanyola del Vallés, Catalonia, Spain.

4 pages, 3 figures.

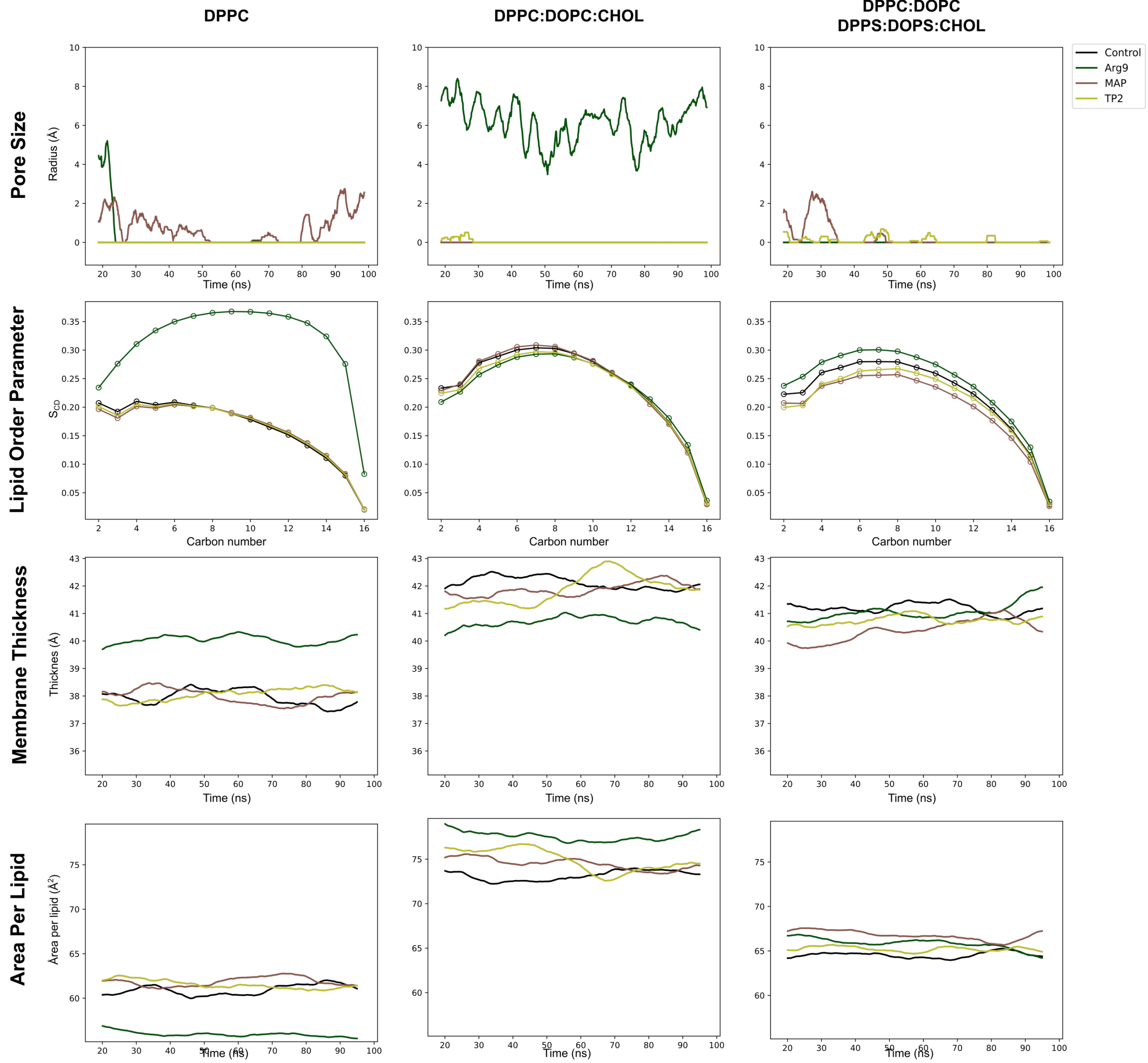

Figure S1. Pore size, lipid order parameter, membrane thickness and area per lipid analyses. The analyses have been performed for the last 80 ns of the cMD part of the DPPC, DPPC:DOPC:CHOL and DPPC:DOPC:DPPS:DOPS:CHOL membrane simulations. Lipid Order Parameter has been computed for the lipid tails and results are shown for carbon number 2 to 16.

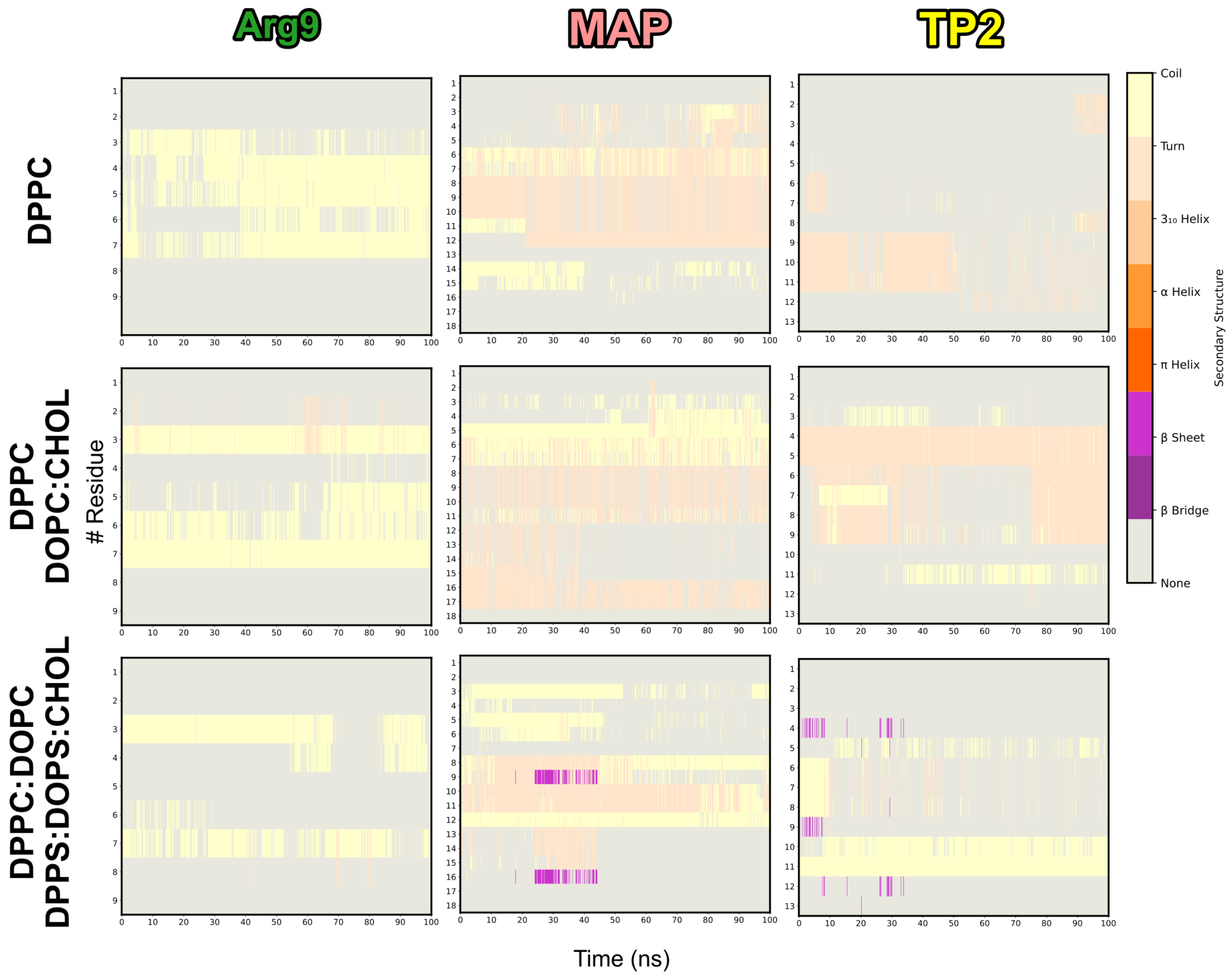

Figure S2. Secondary structure. The secondary structure analysis has been performed for each peptide in the cMD part of the DPPC, DPPC:DOPC:CHOL and DPPC:DOPC:DPPS:DOPS:CHOL membrane simulations.

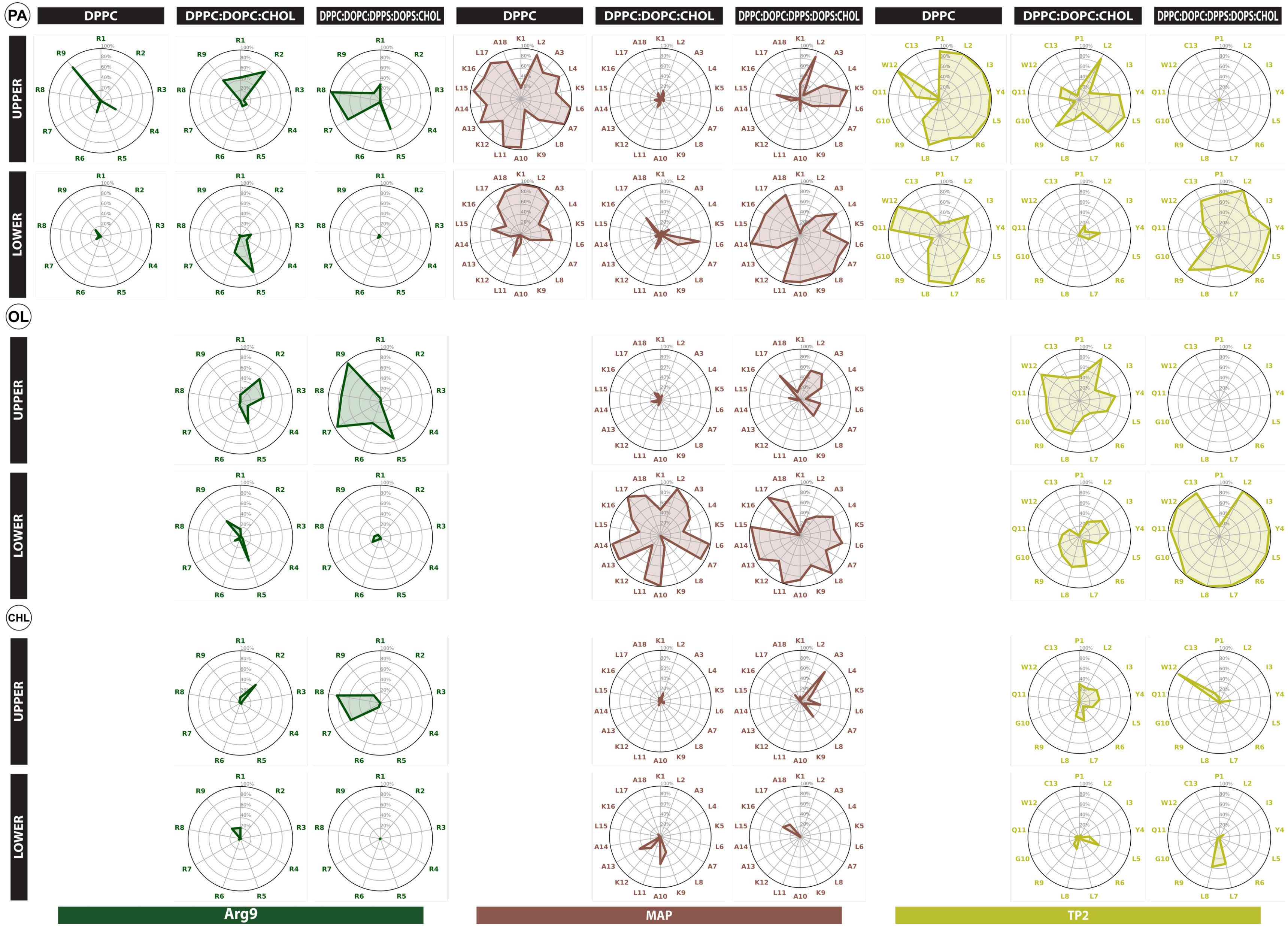

Figure S3. Occupancy of the lipid tails and the cholesterols. PA refers to the lipid tail present in DPPC/DPPS lipids, namely palmitic acid. OL refers to the lipid tail in DOPC/DOPS, namely oleic acid. CHL refers to cholesterol. These are the lipid names provided by the AMBERFF14SB forcefield.
